# Succinylation proteomic analysis identified differentially expressed succinylation sites affecting porcine muscle fiber type function

**DOI:** 10.1016/j.fochx.2023.100962

**Published:** 2023-10-27

**Authors:** Xiaofan Tan, Kaiqing Liu, Yu He, Zhiwei Yan, Jing Chen, Ruixue Zhao, Xin Sui, Junpeng Zhang, David M. Irwin, Shuyi Zhang, Bojiang Li

**Affiliations:** a College of Animal Science and Veterinary Medicine, Shenyang Agricultural University, Shenyang 110866, China; b Shenzhen Institute of Translational Medicine, The First Affiliated Hospital of Shenzhen University, Shenzhen Second People's Hospital, Shenzhen University, Shenzhen 518000, China; c Department of Laboratory Medicine and Pathobiology, University of Toronto, Toronto, Ontario M5S 1A8, Canada

**Keywords:** Pigs, Muscle fiber type, Succinylation proteomic, Differentially expressed succinylation sites, Meat quality

## Abstract

Muscle fiber type is a major factor in pork meat quality, however, the role of post-translational protein modifications, especially succinylation, in the regulation of muscle fiber type is not fully understood. Here we performed protein succinylation profiles of fast-type biceps femoris (BF) and slow-type soleus (SOL) muscles. A total of 4,221 succinylation sites were identified from these samples, of which 294 sites were differentially expressed. Gene Ontology (GO) and Kyoto Encyclopedia of Genes and Genomes (KEGG) enrichment analyses showed that these succinylated proteins were mainly involved in glycolysis, tricarboxylic acid cycle, and fatty acid metabolism. Succinylation modification of the CRAT and RAB10 proteins was verified by co-immunoprecipitation. Protein-protein interaction (PPI) network analysis unveiled the interactions of these succinylated proteins that regulate pig myofiber type conversion. This investigation offers fresh perspectives into the molecular roles of protein succinylation in the regulation of pig myofiber type transformation and meat quality.

## Highlights

• We performed protein succinylation profiles between BF and SOL samples.
• A total of 294 differentially expressed succinylation sites (DESSs) were identified.
• The proteins containing DESSs were involved in the glycolysis, tricarboxylic acid cycle (TCA).
• A PPI analysis was constructed based on proteins containing DESSs.

## 1 Introduction

Muscle fiber composition determines the contractive and metabolic capacity of skeletal muscle, exerting a significant influence on the meat quality in livestock (Sawano and Mizunoya, 2022). Skeletal muscle fibers can be classified into four distinct types due to variation within the myosin heavy chain (MyHC): MyHC I (slow oxidation type), MyHC IIA (fast oxidation type), MyHC IIX (fast oxidation glycolytic type), and MyHC IIB (fast glycolytic type) (Kim et al., 2016). Meat quality, including color, water retention, marbling, and meat texture, is influenced by the characteristics of its muscle fibers (Lefaucheur, 2010). In recent years, the demand by consumers for higher-quality meat has increased. Several studies have proposed that transitioning muscle fibers from type IIB to type I should result in meat that is more tender, thus, enhancing meat quality (Chen et al., 2022, Genxi et al., 2014). A study has shown that the microRNA miRNA-23a significantly reduced the abundance of slow myosin heavy chain subtypes affecting meat quality through a reduction of MEF2C mRNA and protein levels (Shen et al., 2016b). Zhang *et al*. found that butyrate regulates slow muscle fiber formation and mitochondrial biosynthesis through microRNAs and PGC-1α, ultimately improving meat quality (Zhang et al., 2019). Cao *et al*. revealed that a circRNA, circMYLK4, regulates fast/slow muscle fibers in porcine skeletal muscle (Cao et al., 2022). Elevated levels of circMYLK4 were correlated with a remarkable increase in both mRNA and protein levels of genes associated with slow muscle characteristics, thereby promoting slow muscle fiber development (Cao et al., 2022). A recent study highlighted that a balance of branched-chain amino acids plays a pivotal role in governing meat quality by regulating muscle fiber type conversion and intramuscular fat accumulation in finishing pigs (Zhang et al., 2022). Consequently, a more comprehensive comprehension of the mechanisms regulating the transformation of muscle fiber types is imperative for improving meat quality. Nevertheless, the intricate molecular mechanisms underlying the development of muscle fiber types in pigs remain incompletely elucidated.

Post-translational modifications (PTM) refer to the introduction of chemical molecules onto proteins, enabling the modulation of the activity and/or function of these proteins (Bradley, 2022). Lysine succinylation is a prevalent PTM in eukaryotic and prokaryotic cells and this modification significantly influences various metabolic processes, underscoring its vital regulatory role (Shen et al., 2016a). A study has highlighted the wide presence of lysine succinylation across a diverse array of mitochondrial metabolic enzymes (Park et al., 2013). These enzymes play crucial roles in fatty acid metabolism, amino acid degradation, and the tricarboxylic acid cycle (TCA) (Park et al., 2013). Presently, investigations into succinylation predominantly center around its implications in various diseases, including Alzheimer’s disease (Yang et al., 2022a). A previous study characterized changes in succinylation sites in protein from mouse embryonic fibroblasts after SIRT5 knockout (Park et al., 2013). There are limited number of studies focusing on the role of succinylation modifications in meat quality and muscle development. It remains unclear whether succinylation modification is associated with pig muscle fiber type.

Previous studies have reported a greater abundance of slow-type muscle fibers, and better meat quality, for soleus (SOL) muscle compared to biceps femoris (BF) muscle (He et al., 2022, Jin et al., 2021). This study comprehensively analyzed the succinylation patterns of proteins derived from these two distinct muscle types. By examining three muscle samples from each type, the study identified and characterized succinylated proteins and detected the locations of the succinylation modification proteins. GO and KEGG functional analyses of the succinylated proteins were carried out. A subsequent investigation was conducted of the distinct succinylation sites that exhibited differential expression between SOL and BF muscles. GO and KEGG enrichment analyses were performed based on these proteins with differentially expressed succinylation sites (DESSs). Ultimately, a regulatory protein–protein interaction (PPI) network was established for proteins with DESSs. This study establishes the groundwork for understanding the role of protein succinylation modification in forming pork muscle fiber types and meat quality.

## 2 Materials and methods

### 2.1 Animals and sample collection

The biceps femoris (BF) and soleus (SOL) samples used in this study were taken from three male full-sib offspring (106.17 ± 0.76 kg) from a Duroc boar and Meishan sow as described in a previous study (He et al., 2022). These animals were reared under uniform conditions for a duration of 180 days prior to the slaughtering process and were slaughtered in a standardized commercial abattoir (Huaian, China) as per Chinese slaughter guidelines (GB/T 17236–2019). Subsequently, the collected samples were frozen using liquid nitrogen and preserved at −80 °C for further use. Ethical approval for all animal-related procedures was granted by the Ethics Committee and Experimental Animal Committee of Shenyang Agricultural University (Approval number 202006032).

### 2.2 Protein extraction and digestion

Muscle tissue samples were lysed, and proteins were extracted with urea buffer (8 M urea, 100 mM Tris/HCl, pH 8.5). The concentration of proteins was assessed utilizing a Bradford Kit (Beyotime, Shanghai, China), as per the provided guidelines. Proteins were separated on 12.5 % SDS-PAGE gel and visualized through Coomassie blue R-250 staining. For each sample, a final concentration of 10 mM dithiothreitol (DTT) was added and mixed at 600 rpm for 1.5 h at 37 °C, followed by cooling to room temperature. Subsequently, each sample was added a final concentration of 50 mM Iodoacetamide (IAA), and the mixture was incubated in darkness for 30 min. Trypsin was then added at a trypsin-to-protein ratio of 1:50 (wt/wt) and incubated overnight at 37 °C. Post digestion, Trifluoroacetic acid (TFA) was added to each sample to attain a final concentration of 0.1 %, effectively adjusting the pH value to below 3. Digested peptides were desalted from each sample utilizing C18 cartridges (Empore™ SPE cylinder C18, bed ID 7 mm, volume 3 ml), followed by freeze-drying to facilitate subsequent processing.

### 2.3 Enrichment of succinylated peptides

A PTMScan ® Succinyl-Lysine motif kit (Cell Signaling Technology, MA, USA) was used to enrich the samples for succinylated peptides. In brief, lyophilized peptides were redissolved in precooled immunoaffinity purification (IAP) buffer and subsequently subjected to incubation with pretreated anti-succinyl-lysine antibody beads at 4℃ for 1.5 h. Following the incubation, the samples were centrifugated at 2000 × g for 30 s, and the supernatant was discarded. Moreover, the anti-succinyl-lysine antibody beads were then washed three times using pre-chilled IAP buffer, followed by three additional washes with pre-chilled water. Afterward, these beads were incubated with 0.15 % TFA at room temperature for 10 min, centrifugated at 2000 × g for 30 s and the resulting supernatant was desalted using C18 stage tips.

### 2.4 Liquid chromatography-tandem mass spectrometry (LC-MS/MS) analysis

LC-MS/MS analysis was carried out on a Q Exactive HF/HFX mass spectrometer (Thermo Scientific, MA, USA) coupled to an Easy nLC (Thermo Fisher Scientific, MA, USA) for the duration of 120 min. The procedure involved loading samples onto a reverse phase trap column (Acclaim PepM. ap100 C18, 100 μm × 2 cm; Thermo Scientific) connected to the C18-reversed phase analytical column (3 μm, 75 μm × 10 cm; Thermo Scientific) in buffer A (0.1 % Formic acid). Separation was achieved through a linear gradient of buffer B (84 % acetonitrile and 0.1 % Formic acid) at a flow rate of 300 nl/min. The mass spectrometer (MS) was set to functionate in the positive ion mode. The MS data were collected through a data-dependent top10 approach, dynamically selecting the most abundant precursor ions from the survey scan within the 300–1800 *m*/*z* range for higher-energy collisional dissociation (HCD) fragmentation. The automatic gain control (AGC) target was established as 3 × 10^6^, while the maximum inject time was set at 10 ms. A dynamic exclusion duration of 40.0 s was implemented. Survey scans were captured at a resolution of 70,000 at *m*/*z* 200, whereas the resolution for the HCD spectra was set to 17,500 at *m*/*z* 200, accompanied by an isolation width of 2 *m*/*z*. For HCD fragmentation, a normalized collision energy of 30 eV was employed, and an underfill ratio of 0.1 % was specified.

### 2.5 Succinylated proteome data analysis

Raw MS data for each sample was searched against the pig UniProt proteome database (122175 sequences, downloaded January 2022) using the MaxQuant software (Cox et al., 2011) for the identification and quantitation analysis. The specified parameter settings were as follows: the enzyme used was trypsin; a maximum of 2 missed cleavages was allowed; the precursor mass tolerance was set to 20 ppm; and MS2 mass tolerance was also set at 20 ppm. Variable modifications included oxidation (m), and succinyl (k), while carbamidomethylation served as a fixed modification. This study retained peptides, sites, and proteins with less than 1 % FDR. Succinylation sites with fold changes >2.0 or <0.5 and *P-*values < 0.05 were considered to be DESSs.

### 2.6 Motif analysis of succinylation sites

Motifs were subjected to analysis using MeMe (https://meme-suite.org/index.htm). For this analysis, amino acid sequences encompassing the modified site along with six amino acid residues upstream and downstream (13 amino acid sites in total), were utilized as the input for the motif analysis.

### 2.7 Protein subcellular localization and domain analysis

The CELLO (http://cello.life.nctu.edu.tw/), which employs a multi-class SVM classification system, was employed for the prediction of subcellular localization of protein. To identify protein domains, InterProScan software was utilized to align the protein sequence with the Pfam database (Finn et al., 2016).

### 2.8 GO and KEGG analysis of succinylated proteins

Sequences homologous to the succinylated proteins were identified utilizing NCBI blast + client software (NCBI-blast-2.2.28+ -win32.exe) and InterProScan software. Furthermore, the succinylated proteins were subjected to a GO term annotation utilizing the Blast2GO software program (Gotz et al., 2008). To facilitate KEGG annotation, the succinylated proteins were subjected to a BLAST search against the KEGG database (https://www.kegg.jp). A Fisher's exact test was used to assess the GO and KEGG enrichment analyses, with a p-value of less than 0.05 for GO and KEGG considered to be significantly enriched.

### 2.9 Protein-protein interaction analysis

A protein-protein interaction network was constructed using the STRING database (https://cn.string-db.org) with default parameters. The PPI regulatory network was visualized using Cytoscape software.

### 2.10 Protein extraction, co-immunoprecipitation (Co-IP) and western blot analysis

Muscle tissue samples were lysed utilizing RIPA lysate buffer (Beyotime, Shanghai, China) supplemented with PMSF. After lysis, the lysate was centrifuged at 4℃ at 12,000 × g for 15 min, and the resulting supernatant carefully collected into a fresh tube. Furthermore, protein concentration was measured with a BCA kit (Sangon Biotech, Shanghai, China). Subsequently, 1 mg of protein lysate was incubated overnight at 4 °C with 1 μg of the respective antibody (CRAT, RAB10 or IgG; Proteintech, Wuhan, China). Protein A/G magnetic beads were thoroughly washed using binding/wash buffer (0.05 % Tween-20 TBS containing 0.15 M NaCl). These beads were introduced into the antigen sample and antibody mixture, and then incubated at 37 °C for 1 h while ensuring mixing. Subsequently, the magnetic beads were washed three times with PBS, followed by a wash with ultra-pure water. Next, 5 × SDS (250 mM Tris-HCl, 10 % SDS, 50 % glycerin, 5 % β-hydrophobic ethanol) was added and the samples were boiled at 95 °C for 10 min. Supernatants were then collected for subsequent western blot experiments.

The denatured proteins were separated by electrophoresis using a FuturePAGE^TM^ protein gel at 150 V for 60 min during electrophoresis. After electrophoresis procedure, the proteins were transferred onto a PVDF membrane (Millipore, Corp, Bedford, MA). The PVDF membrane was initially blocked with a protein-free rapid-blocking buffer (Shanghai EpiZyme Biotechnology, Shanghai, China) for 15 min, washed three times with TBST and subsequently incubated with primary antibodies for CRAT or RAB10 (Proteintech, Wuhan, China) or Succinyl-lysine (PTM biolabs, Hangzhou, China) overnight at 4℃. Membranes were then washed three times with TBST, and incubated with HRP-conjugated secondary antibodies (Proteintech, Wuhan, China) at room temperature for 1.5 h, followed by three washes with TBST. Protein bands were visualized using an ECL Chemiluminescence Kit (Beyotime, Shanghai, China) and protein abundance was quantified using ImageJ software (National Institutes of Health, Bethesda, MD, USA).

### 2.11 Statistical analysis

All statistical analyses were performed using GraphPad Prism 9.0.0 software (GraphPad Software, La Jolla, USA). Significance of differences between pairs of groups was evaluated utilizing the student *t-*test. The results were displayed as the mean ± SEM. A *P-*value of less than 0.05 was considered to be a statistically significant difference.

## 3 Results

### 3.1 Global succinylation proteomic analysis of BF and SOL muscle tissue

**Fig. 1:**
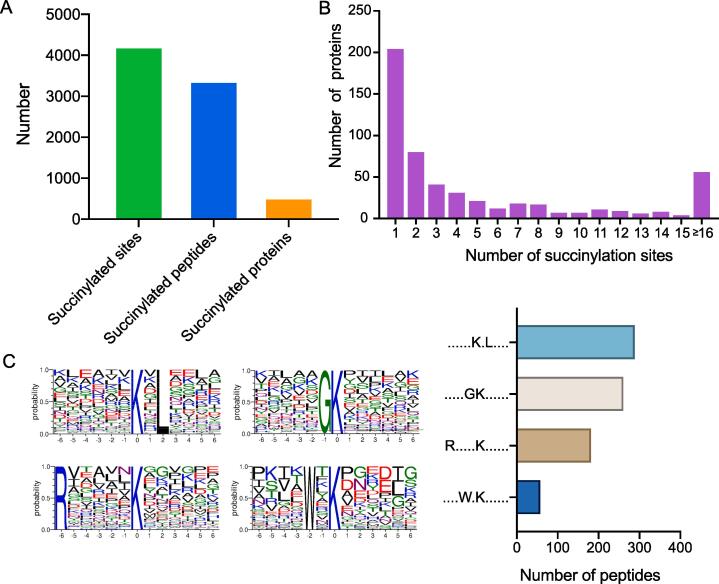
Succinylome analysis of pig BF and SOL muscle tissues. (A) Overview of the number of identified succinylated sites, peptides, and proteins in this study. (B) Count of succinylation sites within individual proteins. (C) Motif analysis of the six amino acid residues upstream and downstream of the identified succinylation sites. The height of each letter indicates the probability of the respective amino acid being present at that specific position. The central amino acid denotes the succinylation site itself. The bars represent the number of each peptide motif.

To explore the functional impact of the protein succinylation modification on fast-type (BF) and slow-type (SOL) muscles, this study characterized the succinylation profiles of proteins from these tissues by 4D label-free quantitative proteomics. This analysis identified 4,221 sites that are succinylated, contained within 3,382 peptides and 532 proteins (Fig. 1A and Table S1). A statistical examination of the number of succinylation sites in these proteins revealed that 61.65 % of the proteins possess two or more succinylation modification sites. However, we still identified more than 200 proteins that had a solitary succinylation modification site (Fig. 1B). A motif analysis, focusing on the adjacent upstream and downstream amino acid residues, was conducted for the identified succinylation modification sites. The major lysine succinylation motifs are K × L, GK, R×××××K, and W × K. Specifically, glycine(G), arginine (R), and tryptophan (W) were found to be preferred at an upstream site of the lysine succinylation site, while leucine (L) was preferred downstream. Among these motifs, the K × L motif was the most frequently observed succinylation modification site motif (Fig. 1C).

### 3.2 Functional analysis of proteins with succinylation modification

**Fig. 2:**
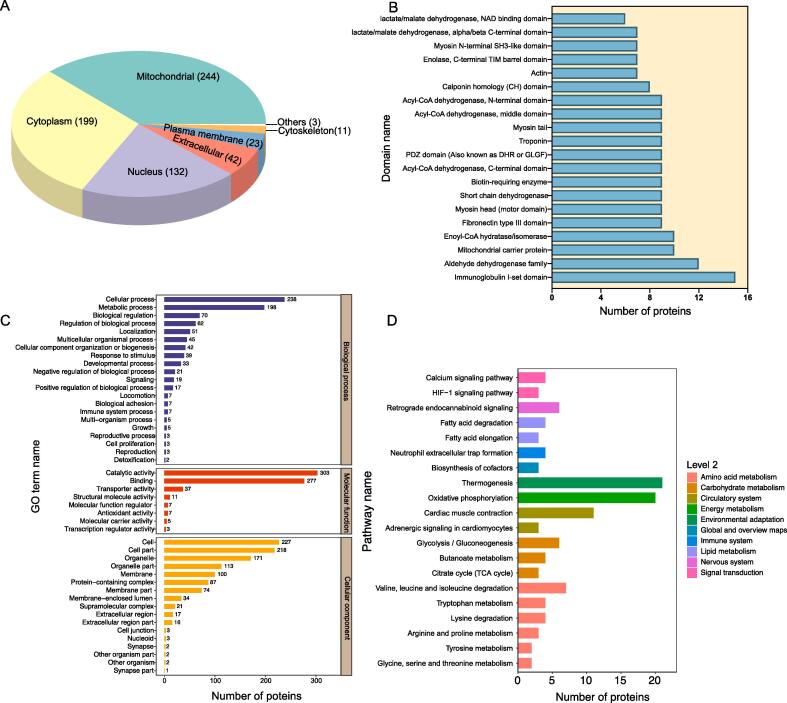
Functional analysis of the succinylated proteins. (A) Subcellular distribution of the succinylated proteins. (B) Protein domain analysis of the identified succinylated proteins. (C) and (D) Representative GO and KEGG pathway annotation of the identified succinylated proteins.

To gain insight into the potential functional involvement of succinylated proteins in muscle tissue, the CELLO software was employed to predict the subcellular localization of each succinylated protein. Succinylated proteins identified in this study were observed to be distributed across various cellular compartments. Specifically, 244 proteins were predicted to localize to the mitochondria, 199 to the cytoplasm, and 132 to the nucleus (Fig. 2A). Some proteins were predicted to have an extracellular localization (Fig. 2A). Protein domains in the succinylated proteins were predicted using InterProScan software. The results revealed that the immunoglobulin I-set protein domain was the most abundant domain among the identified proteins (Fig. 2B and Table S2). Furthermore, a comprehensive GO functional annotation was conducted for all identified succinylated proteins in this investigation, resulting in 2,892 GO terms being annotated to these proteins, of which 1,963, 305, and 624 belonged to biological process (BP), cellular component (CC), and molecular function (MF) categories, respectively (Table S3). The annotation “catalytic activity” predominated as the most frequent in the MF category, followed by proteins annotated as “binding” (Fig. 2C). In the BP category, many proteins were associated with “cellular processes” and “metabolic processes” (Fig. 2C). For CC, most proteins were annotated in “cell” and “cell part” (Fig. 2C). In addition, the KEGG annotation of these succinylated proteins divided them into 78 distinct pathways. Among these, a substantial number of proteins were involved in pathways such as “thermogenesis” and “oxidative phosphorylation” (Fig. 2D and Table S4).

### 3.3 Detection of differentially expressed succinylation sites (DESSs) between BF and SOL muscles

**Fig. 3:**
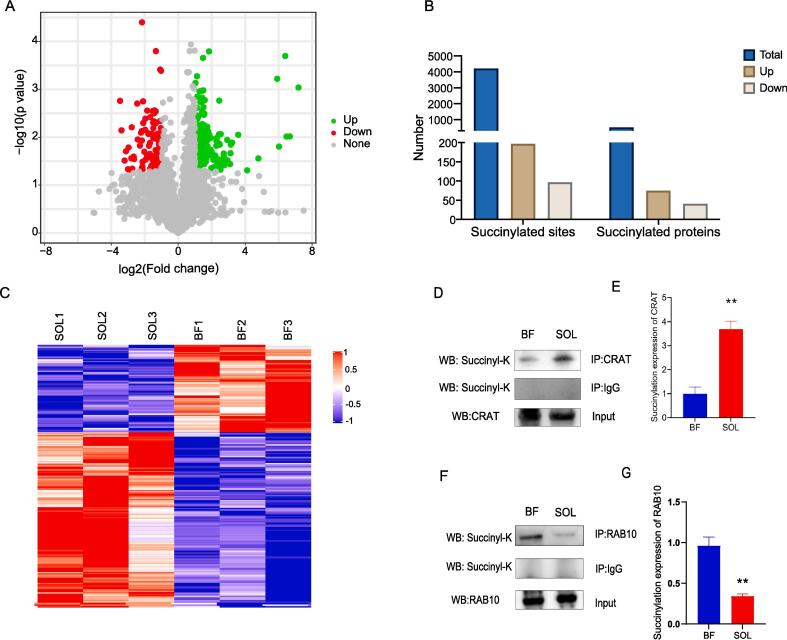
Identification and validation of DESSs in BF and SOL muscle tissues. (A) Volcano plot showing DESSs in BF vs. SOL. The x-axis represents the log2 (fold change) of DESSs between BF and SOL, and the y-axis represents the -log10 (*P*-value). The green, red, and gray dots represent upregulated, downregulated, and no-change DESSs. (B) The number of the total, up-, and downregulated DESSs and their corresponding proteins. (C) Heatmap showing the cluster analysis of the succinylated peptides in BF vs. SOL group. (D) and (F) Succinylation levels of CRAT and RAB10 in BF and SOL tissue samples, respectively. Immunoprecipitation was first performed with CRAT and RAB10 antibodies, respectively, followed by a western blot using an anti-succinylated antibody. (E) and (G) Quantification of the succinylation levels of CRAT and RAB10 protein, respectively. ** represents the *P-*value less than 0.01. (For interpretation of the references to color in this figure legend, the reader is referred to the web version of this article.)

To determine whether protein succinylation may have a role in muscle fiber type transformation, we identified the differentially expressed succinylation sites (DESSs) between BF and SOL muscle tissues. The identified DESSs are shown in Fig. 3A. When levels of succinylation at sites were compared between BF and SOL muscle tissues, 294 DESSs were detected. Among these, 197 sites corresponding to 75 proteins exhibited an upregulation, while 97 sites from 41 proteins exhibited a downregulation **(**Fig. 3B). Additional details on these DESSs are provided in Table S5. Noteworthy, the top three upregulated succinylated sites were K312 in Aldehyde dehydrogenase 6 family member A1 (ALDH6A1), K2 in A0A4X1UD45 (uncharacterized protein), and K1790 in Myosin-4 (MYH4). Conversely, the top three downregulated succinylated sites were K306 in Calcium uniporter protein (MCU), K34 in Quinone oxidoreductase (CRYZ), and K123 in 4HBT domain-containing protein. A heatmap that visually represents the patterns of the identified DESSs in SOL and BF muscles is shown in Fig. 3C, which suggests a potential association of some of these DESSs with muscle fiber type transition.

To confirm the differential expression of succinylation sites, we used Co-IP and western blot analysis to measure succinylation levels of two proteins, CRAT and RAB10, containing a single DESS in BF and SOL muscle tissue. These analyses showed that the succinylation level of CRAT protein was substantially higher in SOL tissue in contrast to BF tissue, whereas the succinylation level of RAB10 was significantly down-regulated (Fig. 3D – G). The agreement between the Co-IP/western blot analysis and the proteomic findings for these two proteins supports the reliability of the identification of DESSs in this study.

### 3.4 Functional enrichment analysis of proteins containing DESSs

**Fig. 4:**
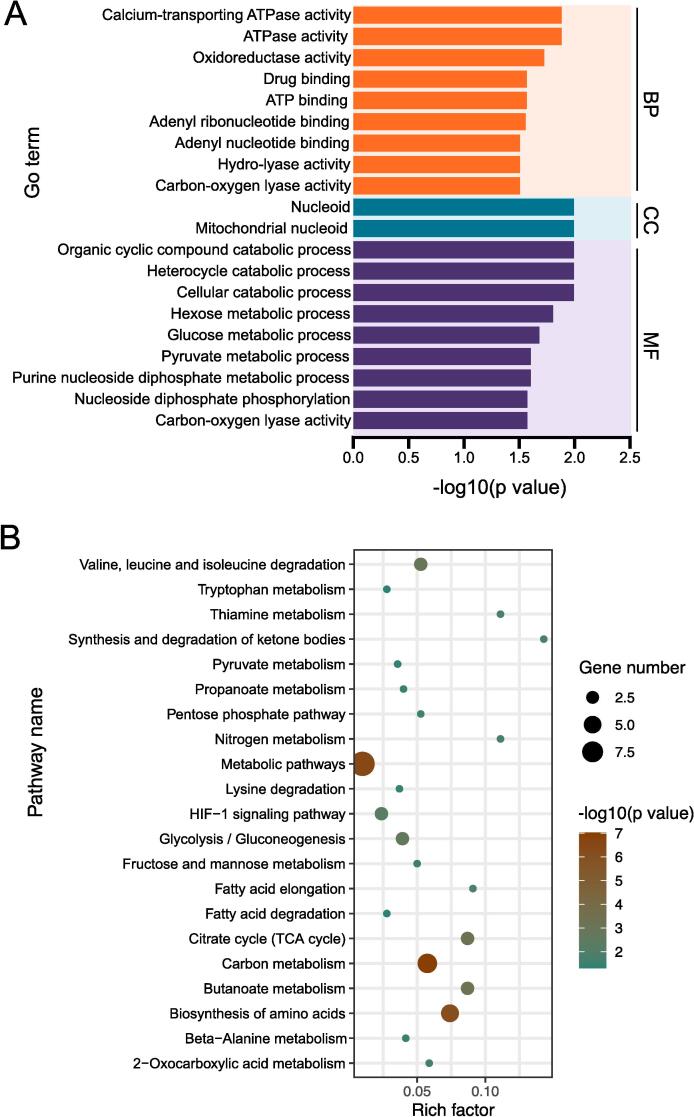
GO and KEGG enrichment analyses of proteins with DESSs. (A) Top 20 significantly enriched GO terms in biological process (BP), cellular component (CC), and molecular function (MF). The x-axis represents the *P*-value, and the y-axis represents the name of the GO term. (B) Significantly enriched pathways. The x-axis represents the rich factor, and the y-axis represents the pathway. The color and size of the dots represent s log10 (*P-*value) and the number of proteins, respectively.

To comprehensively understand the functional roles of proteins containing DESSs between muscle tissue with different fiber types, we conducted GO and KEGG enrichment analyses of the proteins harboring these DESSs. The results of the GO analysis yielded significant enrichments of 63 GO terms, with two GO terms in the CC category, 20 in the MF category, and 41 in the BP category (Table S6). The top 20 notably enriched GO terms are visually depicted in Fig. 4A. Interestingly, many of the GO terms are associated with the transformation and metabolism of muscle fibers, encompassing diverse aspects, including “ATPase activity” in BP, “mitochondrial nucleoid” and “nucleoid” in CC, and “hexose metabolism process”, “glucose metabolism process”, and “pyruvate metabolism process” in MF (Fig. 4A and Table S6). The KEGG enrichment analysis was performed on the proteins containing DESSs, revealing 21 significantly enriched pathways (Fig. 4B). Details on the enriched pathways are shown in Table S7. The most enriched pathways are metabolic pathways, including some pathways of relevance to muscle fiber types such as the “HIF-1 signaling pathway”, “glycolysis/gluconeogenesis”, and “citrate cycle (TCA cycle)” (Fig. 4B), which agrees with our previous research on proteins with differentially expressed phosphorylation sites (He et al., 2022).

### 3.5 PPI analysis of proteins containing DESSs

**Fig. 5:**
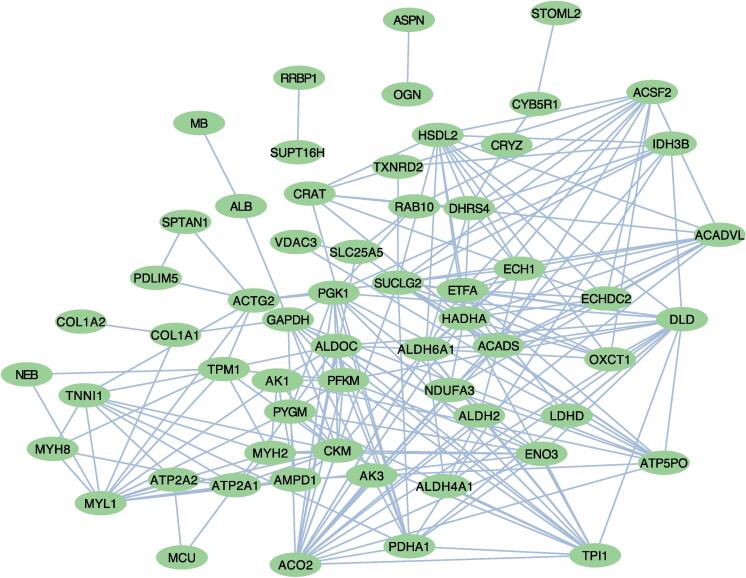
PPI regulatory network analysis based on proteins with DESSs. Ellipses represent succinylated proteins. The lines identify proteins that interact with each other.

To further investigate whether succinylated proteins might play a role in regulating the conversion of muscle fiber types through intermolecular binding, a protein–protein interaction (PPI) regulatory network was constructed using these proteins containing DESSs. Our PPI network contained 60 succinylated proteins and had 219 identified interactions (Fig. 5 and Table S8). The network suggested that CKM is a potentially pivotal protein for regulating the transformation of muscle fiber types through succinylation modifications. This proposition stems from its association with 15 other proteins containing DESSs. Intriguingly, key glycolytic enzymes such as ENO3, GAPDH, PFKM, and TPI1, are among these interacting proteins, underpinning the significance of the potential regulatory role of CKM.

## 4 Discussion

Lysine residues are essential for shaping protein structures and facilitating their functions. Succinylation of lysine residues in proteins is a prevalent post-translational modification found in both eukaryotes and prokaryotes (Weinert et al., 2013). Succinylation entails the transfer of a succinyl group from the succinyl donor molecule, succinyl-CoA, to a specific lysine residue located within a protein (Li et al., 2020). Zhang *et al.,* in 2011, conducted a comprehensive study using HPLC-MS/MS analysis and further identified lysine succinylation modification (Zhang et al., 2011). Notably, compared to other PTMs, including phosphorylation, methylation, and acetylation, succinylation causes a change in the protein charge from +1 to −1 at the modified site. Furthermore, this modification leads to a relatively substantial increase in mass (Yang and Gibson, 2019). To date, a large number of succinylation sites have been identified in proteins in animals such as mice (Park et al., 2013). In mouse live cells, 2,565 succinylation sites within 779 proteins were detected (Park et al., 2013). Yang *et al.* identified 1,485 succinylation sites in 568 proteins in human lung tissue by a succinylome analysis (Yang et al., 2022b). In silkworms, 1,884 succinylation sites in 373 proteins were identified, and these succinylation-modified proteins were found to be mainly enriched in central metabolic processes (Chen et al., 2018). However, succinylation profiles have not yet been reported for the pig. Our study successfully identified 4,221 succinylated sites in 532 proteins from BF and SOL muscle tissues in pig. This result significantly contributes to the diversity and quantity of succinylation sites identified in animal muscle proteins. Among the identification of succinylated proteins, the most abundant proteins were localized to the mitochondria, aligning consistently with the results of multiple previous investigations (Fu et al., 2022, Weinert et al., 2013, Yang et al., 2022a). Furthermore, four conserved motifs were discovered, characterized by the leucine, glycine, arginine, and tryptophan residues around the succinylated lysine site. Interestingly, the GK and R××××××K motifs were also observed in succinylated proteins in mouse liver (Park et al., 2013) and in *Vibrio alginolyticus* (Zeng et al., 2020), respectively. This observation suggests that these motifs are potentially conserved in various species.

In this study, a total of 294 DESSs on 109 distinct proteins were identified. This observation indicates that these proteins could potentially play a critical role in porcine muscle fiber type conversion by undergoing succinylation modification. However, the molecular mechanisms of these DESSs in the transformation of muscle fiber types need to be validated in large populations in the future. Prior research has consistently demonstrated that protein succinylation can influence protein stability and activity, ultimately regulating biological function (Ou et al., 2020, Wang et al., 2019). In SIRT5 knockout brown adipose tissue, the succinylation level of the UCP1 protein was upregulated (Wang et al., 2019). Consequently, both the stability and functionality of UCP1 were compromised, contributing to the subsequent inhibition of mitochondrial respiratory processes (Wang et al., 2019). Interestingly, the succinylation levels of critical proteins involved in regulating muscle fiber types was significantly altered in the present study. For example, the fast-type muscle fiber protein, MYH4, is highly succinylated with 34 upregulated succinylation sites in SOL tissue. This data implies that excess succinylation on MYH4 potentially destabilizes it, ultimately leading to a transition from fast to slow muscle. In addition, sarcomeric proteins that form the sarcomeric unit undergo succinylation modifications, with three sites in TNNT3 and one in ATP2A1. These data suggest that highly succinylated MYH4 and sarcomeric proteins promote the transition from a fast to slow muscle fiber type. A previous study revealed that the interaction between FHL1 and NFATC1 stimulates the transcriptional activity of NFATC1, subsequently leading to an enhancement in the expression of genes associated with oxidative muscle fibers (Cowling et al., 2008). In this study, low succinylated levels of FHL1 in SOL muscle suggest that it may contribute to slow-type muscle fiber development. Furthermore, it is important to note that CRAT, a mitochondrial enzyme, has a remarkable connection to skeletal muscle mitochondrial inertia (Hesselink et al., 2022). In this study, CRAT was identified to have a differentially expressed succinylation site, which was verified using a Co-IP experiment. These data suggest that the succinylation of CRAT affects its enzymatic activity, thereby regulating the transformation of myofiber types. Taken together, succinylation modifications modulate the transition between pig muscle fiber types by affecting the activity and stability of proteins.

**Fig. 6:**
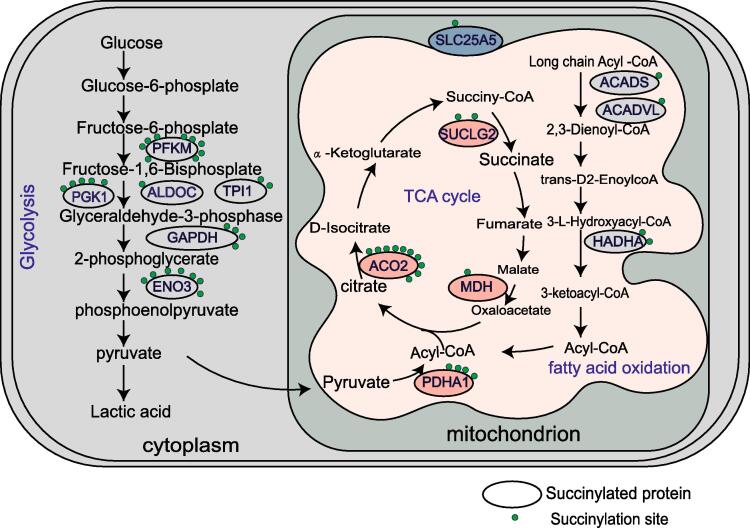
Proteins identified with DESSs are mainly involved in glycolysis, TCA, and fatty acid metabolism. Ellipses represent succinylated proteins. Circles represent succinylation sites on the proteins. These six succinylated proteins are involved in the glycolysis process. PFKM, phosphofructokinase, muscle；PGK1, phosphoglycerate kinase 1；ALDOC, aldolase, fructose-bisphosphate C; TPI1, triosephosphate isomerase 1; GAPDH, glyceraldehyde-3-phosphate dehydrogenase; ENO3, enolase 3. These five succinylated proteins are involved in the TCA. SLC25A5, solute carrier family 25 member 5; SUCLG2, succinate-CoA ligase GDP-forming subunit beta; ACO2, aconitase 2; MDH, malate dehydrogenase; PDHA1, pyruvate dehydrogenase E1 subunit alpha 1. These three succinylated proteins are involved in fatty acid oxidation. ACADS, acyl-CoA dehydrogenase short chain; ACADVL, acyl-CoA dehydrogenase very long chain; HADHA, hydroxyacyl-CoA dehydrogenase trifunctional multienzyme complex subunit alpha.

BF muscle has a higher proportion of type IIB fibers, which are predominantly fast-twitch glycolytic metabolism. On the other hand, SOL exhibits a higher proportion of type I fibers, characterized by their elevated oxidative metabolism levels (Carroll et al., 2011). Additionally, the enrichment analysis of proteins containing DESSs showed that they were enriched in glycolysis and mitochondrial oxidation-related GO terms. Notably, this enrichment included such as “hexose metabolism”, “glucose metabolic process”, and “pyruvate metabolism”. Intriguingly, KEGG enrichment analysis revealed that these proteins containing DESSs can be enriched in several pathways such as “glycolysis/gluconeogenesis”, “pyruvate metabolism”, and “citrate cycle (TCA cycle)”. These findings strongly indicate that succinylation plays a pivotal role in modifying proteins involved in glycolysis and the TCA cycle, ultimately regulating the development of muscle fiber types. Further studies revealed that many key enzymes in glycolysis, TCA, and fatty acid metabolism undergo succinylation modifications (Fig. 6). In glycolysis, PFKM, ALDOC, TPI1, PGK1, GAPDH, and ENO3 enzymes have differentially expressed succinylation sites, with PFKM, in particular, having the most sites (Fig. 6). Previous studies have demonstrated that succinylation of proteins alters the activity of TCA-associated enzymes to regulate aerobic respiration (Liu et al., 2022a, Ou et al., 2020). For instance, PFKM exhibited ten upregulated succinylation modification sites in the SOL muscle. Thus, succinylation modifications inhibit these enzyme activities, thereby promoting the transition from fast to slow muscle type. It is well known that muscles of the slow type are distinguished by their higher mitochondrial content and primarily engage in aerobic metabolism. In contrast, fast-type muscles rely primarily on anaerobic and glycolytic ATP production (Kivelä et al., 2016). Succinylation-modified proteins in the present study were enriched in mitochondria-associated GO terms, including “mitochondrial nucleoid” and “mitochondrial calcium ion homeostasis”. Recent research has indicated that the HIF-1 pathway regulates cell survival and glycolysis-related genes, consequently influencing pig meat quality (Liu et al., 2022b). Additionally, oxidative metabolism was significantly increased in the skeletal muscle of HIF-1α knockout mice (Mason et al., 2004). Our study shows that proteins containing DESSs are significantly enriched in the HIF-1 signaling pathway, suggesting that these proteins potentially regulate muscle fiber types through influencing glycolysis and oxidative metabolism processes.

Prior research has demonstrated that succinylation affects protein-protein interactions (Li et al., 2020, Wang et al., 2022). For instance, Li *et al.* highlighted that the succinylation of L222 in the LDHA protein suppresses its interaction with the SQSTM1 protein, consequently decreasing its degradation (Li et al., 2020). Similarly, the K672 and K799 sites of FBN1 were found to be hypersuccinylated in cancer cells, which prevents its binding to MMP2 (Wang et al., 2022). The PPI regulatory network in our study demonstrated that TNNI1 can bind with ATP2A2 and PDHA1. Notably, ATP2A2 was primarily expressed at elevated levels in slow-type muscles, where its primary role involves the uptake of cytoplasmic calcium ions (Wei et al., 2015). Thus, downregulation of succinylation levels of TNNI1 in SOL may be beneficial for binding to ATP2A2 to promote a fast to slow muscle conversion. Moreover, CKM is situated within the M−band of the sarcomere and expressed predominantly in fast-type muscle fibers (Drexler et al., 2012). In the PPI results, CKM can bind to numerous essential glycolytic enzymes, such as TPI1, ENO3, PFKM, GAPDH, and ALDOC. Intriguingly, CKM was highly succinylated at sites K25 and K172 in SOL muscle tissues in this study. Consequently, these findings propose that a high level of succinylation of CKM inhibits its binding to these enzymes, downregulating the glycolytic process. The PPI lays the foundation for forthcoming investigations on succinylated proteins that might regulate the transformation of pig myofiber types through interactions. Future studies, however, are needed to verify the specific molecular regulatory mechanisms for these interactions.

## 5 Conclusion

Overall, a comprehensive succinylome analysis identified 294 DESSs in 109 proteins between the BF and SOL muscle tissues in pigs. The proteins containing these DESSs are enriched in glycolysis- and TCA-related GO terms and signaling pathways. Succinylation modifications may regulate myofiber transformation by altering the level and activity of key enzymes involved in glycolysis, TCA, and fatty acid metabolism. These findings offer novel insights into the intricate regulation of pig muscle fiber types and provide ways to enhance meat quality by regulating protein succinylation.

## CRediT authorship contribution statement

**Xiaofan Tan:** Methodology, Validation, Investigation, Writing – original draft. **Kaiqing Liu:** Investigation, Writing – original draft, Funding acquisition. **Yu He:** Software, Formal analysis. **Zhiwei Yan:** Methodology, Investigation, Data curation. **Jing Chen:** Formal analysis, Investigation. **Ruixue Zhao:** Data curation, Writing – review & editing. **Xin Sui:** Validation, Investigation. **Junpeng Zhang:** Investigation, Project administration. **David M. Irwin:** Writing – review & editing. **Shuyi Zhang:** Writing – review & editing. **Bojiang Li:** Conceptualization, Writing – original draft, Writing – review & editing, Supervision, Funding acquisition.

## Declaration of Competing Interest

The authors declare that they have no known competing financial interests or personal relationships that could have appeared to influence the work reported in this paper.

## Data Availability

Data will be made available on request.
